# Overview: Systemic Inflammatory Response Derived From Lung Injury Caused by SARS-CoV-2 Infection Explains Severe Outcomes in COVID-19

**DOI:** 10.3389/fimmu.2020.01626

**Published:** 2020-06-26

**Authors:** Rafael B. Polidoro, Robert S. Hagan, Roberta de Santis Santiago, Nathan W. Schmidt

**Affiliations:** ^1^Ryan White Center for Pediatric Infectious Diseases and Global Health, Department of Pediatrics, Herman B. Wells Center for Pediatric Research, Indiana University School of Medicine, Indianapolis, IN, United States; ^2^Division of Pulmonary Diseases and Critical Care Medicine, Department of Medicine, University of North Carolina, Chapel Hill, NC, United States; ^3^Independent Researcher, Boston, MA, United States

**Keywords:** SARS-CoV2, COVID-19, severe COVID-19, bisphosphonates, inflammatory monocytes, ARDS, renin-angiotensin system, kallikrein-kinin system

## Abstract

Most SARS-CoV2 infections will not develop into severe COVID-19. However, in some patients, lung infection leads to the activation of alveolar macrophages and lung epithelial cells that will release proinflammatory cytokines. IL-6, TNF, and IL-1β increase expression of cell adhesion molecules (CAMs) and VEGF, thereby increasing permeability of the lung endothelium and reducing barrier protection, allowing viral dissemination and infiltration of neutrophils and inflammatory monocytes. In the blood, these cytokines will stimulate the bone marrow to produce and release immature granulocytes, that return to the lung and further increase inflammation, leading to acute respiratory distress syndrome (ARDS). This lung-systemic loop leads to cytokine storm syndrome (CSS). Concurrently, the acute phase response increases the production of platelets, fibrinogen and other pro-thrombotic factors. Systemic decrease in ACE2 function impacts the Renin-Angiotensin-Kallikrein-Kinin systems (RAS-KKS) increasing clotting. The combination of acute lung injury with RAS-KKS unbalance is herein called COVID-19 Associated Lung Injury (CALI). This conservative two-hit model of systemic inflammation due to the lung injury allows new intervention windows and is more consistent with the current knowledge.

## Introduction

Coronaviruses are a large family of enveloped viruses (Coronaviridae) containing a positive-sense single-stranded RNA genome with a nucleocapsid. These viruses cause illness in both mammals and birds. Within humans they cause respiratory tract infections that range from asymptomatic infection to severe disease and death. Some coronaviruses have exhibited increased capacity to infect and spread among humans, with higher lethality, causing diseases such as SARS, MERS, and more recently, COVID-19. The causative virus of COVID-19 is the recently identified, severe acute respiratory syndrome coronavirus 2 (SARS-CoV-2) (1). Depending on a number of currently unknown factors, SARS-CoV-2 infection causes either asymptomatic infections or clinical disease, which ranges from mild to life-threatening disease.

Angiotensin Converting Enzyme 2 (ACE2) serves as the main cell surface receptor, allowing the SARS-CoV-2 virus to invade host cells (2). Transcriptome analysis of infected epithelial cell lines and swab specimens from patients positive for SARS-CoV-2 (qRT-PCR) showed mild increased reads of *ACE2*, suggesting that *ACE2* is an interferon-stimulated gene (3–5). On the other hand, SARS-CoV have been reported to reduce cell surface expression of ACE2 *in vitro* and *in vivo* (6, 7). It is currently unknown how SARS-CoV-2 modulates *ACE2* transcription in the lung. SARS-CoV, and potentially SARS-CoV-2, might impact extracellular ACE2 from normal function via direct blocking during attachment and invasion, intracellular modulation of the Unfolded Protein Response, and decreased stability of *ACE2* transcripts or altered translation resulting in reduced protein levels. ACE2 functions biologically to convert Angiotensin II (vasoconstrictor) into Angiotensin (vasodilator), thus lowering blood pressure. Another function of ACE2 is to decrease levels of Bradykinin, thus affecting the renin-angiotensin system (RAS) and kallikrein-kinin system (KKS) (8, 9). The balance between ACE/ACE2 affects vascular diseases and higher Bradykinin can increase systemic inflammatory processes (10). A review by Tolouian et al. discusses the possible involvement of the RAS-KKS axis in COVID-19 (10). As RAS has prothrombotic effects (11), part of the coagulation syndrome seen in COVID-19 might be due to viruses escaping into the blood stream and affecting ACE2 expression in the entire body. Given the direct participation of ACE2 in both RAS and KKS, it is likely that those systems directly contribute to local and systemic inflammation.

Unlike viral load associated with SARS, MERS, and Influenza (12) current data suggest there is no difference in upper respiratory viral load between moderate and severe cases of COVID-19 in hospitalized patients, with the exception of one study in China (13–15). Most COVID-19 patients have upper respiratory tract viral replication with mild symptoms, including fever and dry cough, and recover without developing further symptoms. It is possible that if SARS-CoV-2 reaches the lower respiratory tract, the higher amount of ACE2 in alveoli results in progression of infection into a more severe disease. Accordingly, a recent study associates a higher viral load in the sputum with severity of COVID-19 (16). About 20% of COVID-19 cases have fever coupled with pneumonia that progresses to true ARDS, whereas some patients develop cytokine storm associated ARDS that may be accompanied by features of the Macrophage Activation Syndrome (MAS)/lymphohistiocytosis (HLH) spectrum (17–19). The family of conditions due to COVID-19 associated cytokine release was recently named Cytokine Storm Syndrome (CSS) by Henderson et al. (20).

Cytokine storm (CS) is known to contribute to the morbidity in patients infected with other coronaviruses (21, 22). There is also a correlation between IL-6, C-reactive protein (CRP) and respiratory failure in COVID-19 (23, 24). Yet, there is reason to think CS is not the only source of morbidity, and we will present evidence supporting this hypothesis. In addition to CS, mounting evidence suggests an acute lung injury loop including RAS-KKS unbalance, release of immature leukocytes from the bone marrow, lung cellular infiltrate, vascular dysfunction, and coagulopathy can contribute to morbidity and mortality in COVID-19.

## Systemic Inflammatory Response Induced by Lung Inflammation

Recently, Wadman et al. provided a detailed perspective regarding the diverse biological systems that display some level of pathology during COVID-19 (25). Besides the lung, pathology has been observed in the liver, kidneys, intestines, brain, heart, and blood vessels. Despite the complexity of this multisystem syndrome, the peer reviewed book chapter “Systemic Inflammatory Response Induced by Lung Inflammation” by Hiraiwa and van Eeden can shed important light guiding us from the lung to the vascular syndrome without contradicting the evidence seen in COVID-19 patients (26).

ARDS caused by SARS-CoV-2 is characterized by a rapidly induced inflammatory response in the lung. The classification of COVID-19 lung damage as ARDS is under scrutiny because the severe hypoxemia is accompanied be a relatively mild decrease in lung compliance (measurement of lung elastic properties), suggesting that other mechanisms than direct parenchymal injury may contribute to the degree of hypoxia. Some advocate that the COVID-19 lung disease is covered by ARDS definition (27), while others divide the lung damage into two phenotypes, ARDS and not-ARDS (28, 29). Classification of COVID-19 lung damage as ARDS and not-ARDS is not trivial and a deeper comprehension of underlying mechanisms of COVID-19 lung damage is critical to define the proper respiratory management of these patients. For example, while there are evidence-based treatment strategies for rigorously defined ARDS (including low tidal volume ventilation, prone positioning, conservative fluid management, and neuromuscular blockade), other mechanisms of hypoxia such as pulmonary thrombi, acute pulmonary vascular disease, or cardiac injury would necessitate completely different treatment strategies. Previous data has shown that both inadequate timing and strategy of artificial ventilation are harmful to patients (30, 31). Consistent with these observations, a case series from New York City (New York, USA) COVID-19 patients, a high mortality rate (88%) was observed in the artificially ventilated patients (32).

**Figure 1:**
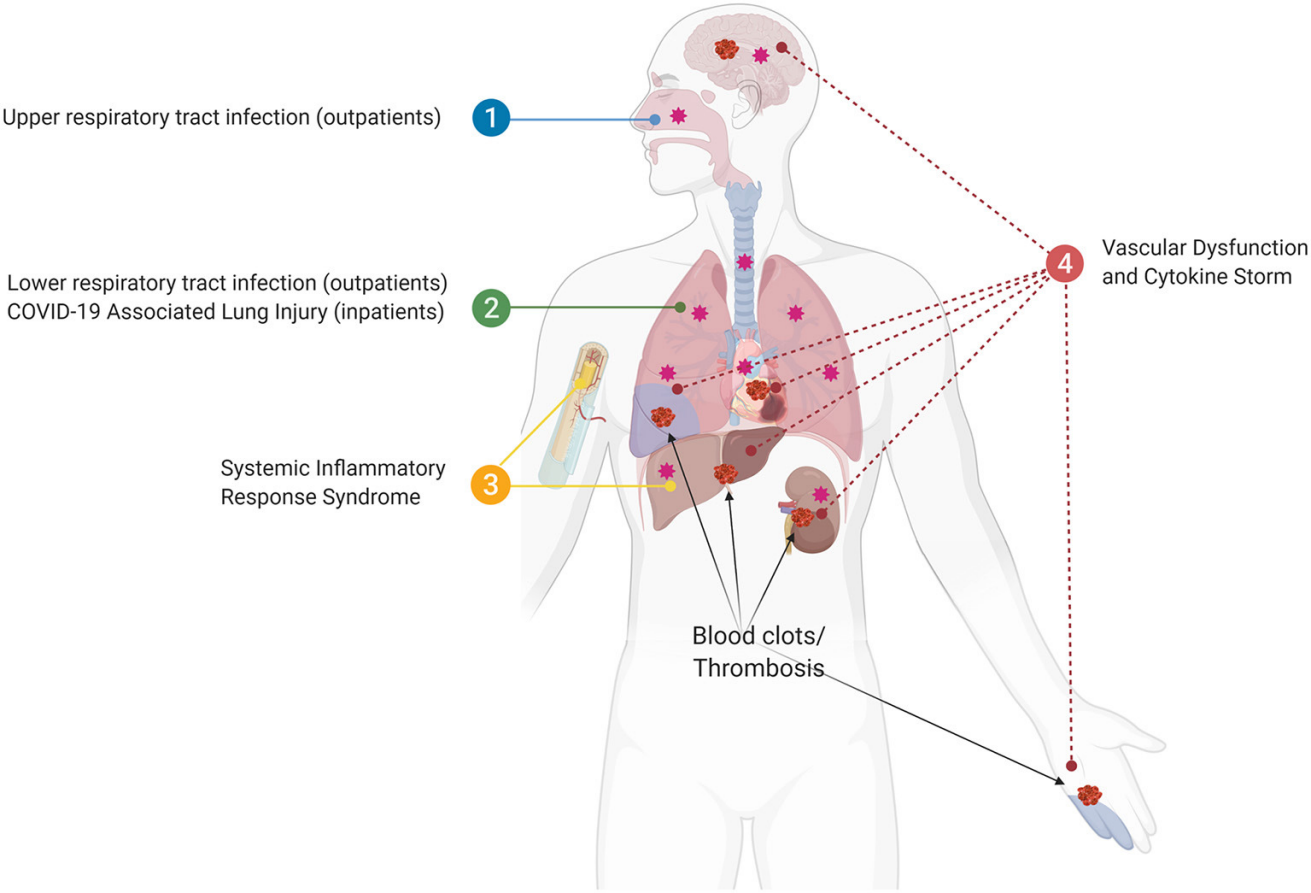
The four phases of SARS-CoV2 infection. (1) Upper and lower respiratory tract infection (outpatients); (2) COVID-19 associated lung injury (CALI), in which some patients will be hospitalized (inpatients); (3) Systemic inflammatory response syndrome (SIRS), in which bone marrow and liver acute phase response accumulate pro-thrombotic factors resulting in blood clots/thrombosis; (4) The sustained loop between lung and systemic inflammation results in multi-organ vascular dysfunction and cytokine storm syndrome.

We propose a view of COVID-19 that is consistent with prior understandings of lung physiology, where local infections progress into systemic pathology associated with exaggerated cytokine production, thrombosis, and multi-organ damage/failure that heighten risk of death (19, 26). Clinical events following severe tissue injury also activate the systemic response of the host in a similar manner to sepsis and the recognition of this common pathophysiologic phenotype led to the term “systemic inflammatory response syndrome” or SIRS (26). To address an overview of how a similar process might be at play with COVID-19 we will divide the progression of SARS-CoV-2 infection into four phases: (1) Upper and lower respiratory tract infection, (2) COVID-19 associated lung injury (CALI; Box 1), (3) SIRS, and (4) systemic failure (Figure 1).

Box 1 **COVID-19 Associated Lung Injury (CALI).**

SARS-CoV-2 appears to induce acute lung injury similarly to other respiratory viruses, but with additional symptoms derived from alterations in the inflammatory resolution phase and RAS-KKS system, possibly due to ACE2 downregulation, and/or elimination of pneumocytes type II (33). The elimination of pneumocytes type II might explain the slow tissue recovery in patients with severe COVID-19.

### Upper and Lower Respiratory Tract Infection

This phase would be the result of the infection itself. Virus infection triggers antiviral innate immunity, resulting in Flu-like symptoms, such as fever, fatigue, body ache, and others. There are many drugs and treatments trying to stop virus multiplication in this and other phases of the disease, some of them are elegantly summarized by Kupferschmidt and Cohen (34). At this point, most infected individuals will recover, and we would expect them to be outpatients. Theoretically, patients would benefit from antivirals as long as virions are present, but we hypothesize the earlier the best to prevent CALI and its progression.

### COVID-19 Associated Lung Injury (CALI)

When the virus reaches the lung, most patients will mount a proper antiviral immune response (outpatients), with higher lymphocyte presence in the lung contributing to viral elimination (35–37). However, in some patients there will be exacerbated local inflammation due to the activation of nucleic acid sensors on lung epithelium and alveolar macrophages (38). This will trigger autocrine and paracrine cytokine release and other proinflammatory mediators, resulting in the recruitment of polymorphonuclear neutrophils, monocytes, and other leukocytes, which have been reported in lungs of patients with COVID-19 (35, 36, 39, 40). Alveolar macrophages and inflammatory monocytes are thought to be a source of IL-6, TGF-β, TNF, IL-8, and IL-1β (38), whereas neutrophils rapidly produce oxygen radicals, lipid mediators, and proteases that can be harmful to viruses, but also to lung tissue (41). Accordingly, single-cell RNA sequencing on bronchoalveolar lavage fluid immune cells from COVID-19 patients has shown the enrichment of macrophages and neutrophils with a strong pro-inflammatory signature on monocyte-derived macrophages, and the severity was associated with much higher levels of *IL8, IL6, TNF*, and *IL1B* (39). Neutrophil extracellular traps (NETs) have been associated with disease severity in COVID-19 (42, 43). A review by Merad and Martin address many details for the role of macrophages and monocytes in the pathology of COVID-19 (44). Following exposure to air pollution particles, alveolar macrophages and epithelial cells increase the production of pro-inflammatory cytokines capable of stimulating the bone marrow, resulting in higher numbers of band cells (i.e., immature granulocytes) in circulation (45). In response to acute inflammation, the liver releases C-reactive protein (CRP) and Serum Amyloid A (SAA) that will increase in the first days whether the patient develops moderate or severe symptoms. Those levels might decrease after 7 days, depending on the resolution of the injury, whereas haptoglobin and fibrinogen (later seen as D-dimer) go up, possibly resulting in blood clots (46). Pro-thrombotic factors are increased in COVID-19 patients since early in the disease and its increase correlates with severe respiratory syndrome (47–49).

Of note, arrested translation due to antiviral immunity and viral manipulation of translational repression would strongly impact protein expression by lung cells (50). ACE2 downregulation impacts the RAS-KKS system resulting in coagulation disturbances, and increases the chances of thrombosis and microthrombosis (10, 11). In malaria, the acute respiratory distress syndrome also presents competitive binding of other surface proteins, such as Endothelial Protein C Receptor (EPCR), that when cleaves its ligand PAR-1, inhibits NFκB activation, reducing overall local inflammation (51). It is currently not known if SARS-CoV-2 infection results in downregulation of those same surface proteins, resulting in higher inflammatory response.

Another aspect of CALI that may occur is the upregulation of endothelial cell adhesion molecules (CAMs) VCAM, ICAM, VWF, ANG-2, and VEGF upon tissue injury. These proteins are upregulated by pro-inflammatory cytokines, proteases, leukotrienes, PAF, and oxidants produced by the neutrophils (26). These factors may culminate in the destruction of the lung glycocalyx, which may increase the permeability of the endothelium, allowing the viruses to pass to the blood stream and reach other organs that express ACE2. This may explain detection of viral particles and RNA in other body fluids (52). Increased permeability of microvascular barriers also results in protein-rich edema fluid in airspaces, resulting in poor gas exchange and decreased blood O_2_ levels, which are a central pathophysiological mechanism in ARDS leading to hospitalizations. Autopsies performed in different centers showed similar findings in lungs from COVID-19 victims: diffuse alveolar disease (DAD) combined with atypical pneumocytes and diffuse thrombosis of small vessels (53–55).

For a proposed clinical-therapeutic staging, please refer to Siddiqi and Mehra (19). Inpatients will most likely present moderate to severe symptoms, possibly hypoxia (defined as PaO_2_/FiO_2_ <300 mm Hg), thrombocytopenia, lymphocytopenia, bilateral pneumonia, ground glass opacity (GGO), with mild elevation of systemic inflammation. Different methods for SARS-CoV2 detection allow inclusion of early biomarkers to assess the quality of the cellular infiltrate and the lung state. Here, we note the potential benefit of the combination of antivirals, vasodilators (e.g., NO), and bisphosphonates for inpatients with risk group comorbidities (Box 2) upon evaluation of potential adverse risks. The idea is to prevent the lung-systemic inflammatory loop in a similar way as fingolimod, a sphingosine 1 phosphate analog that can affect lymphocyte retention in lymphoid organs with a positive outcome reported for COVID-19 (65). The family of bisphosphonates is used in the treatment of osteoporosis, certain bone tumors, and bone metastasis. Bisphosphonates are capable of reducing the number and impairing function of monocytes and specialized macrophages (66). Treatment with a member of this family, clodronate disodium, has shown effectiveness in animal models of pulmonary coronavirus infections (67), but the impact of clodronate disodium on the microglia can also make animals more susceptible to neural damage in a coronavirus encephalitis model (68). Treatment of lung tissues with bisphosphonates during COVID-19 could prevent inflammatory monocytes from exaggerating lung injury. To date, there are no registered clinical trials with bisphosphonates to treat COVID-19. One approach to assess the potential effectiveness of this treatment would be to access COVID-19 databases to address whether osteoporosis or cancer patients under bisphosphonate treatments present better COVID-19 prognostic compared to non-bisphosphonate treated patients. Another study addressing monocytes/macrophages used Diminazene Aceturate (DIZE), which is FDA approved for animal use. DIZE treatment can ameliorate liver fibrosis in mice through reducing ROS generation and pro-fibrotic cytokines by Kupffer cells (69), which are liver-resident macrophages. Additionally, DIZE treatment has also been shown to alleviate lung injury in mice by regulating the ACE2-Ang (1, 7)-Mas axis (70). Reduction of alveolar macrophages with bisphosphonates, DIZE, or other approaches might have negative effects in the resolution of ARDS. Yet, we were not able to find significant drawbacks on respiratory disorders on patients under bisphosphonate treatments within the 70 Clinical Trials that have used bisphosphonate treatment (71). Moreover, studies addressing lung cancer present potential beneficial effects (72).

Box 2 **Risk groups.**

The models of systemic inflammatory response induced by lung injury strongly suggest that lung inflammation is a “two-hit” model. Meaning that underlying inflammation, either caused in the lung, or coming from another site (e.g., cardiovascular diseases, obesity, diabetes, and liver disease) may feedback into the lung upon a new infection like SARS-CoV2, resulting in the exacerbation of the local and systemic inflammation. The RAS-KKS system is also already affected in all those risk groups conditions, including aging (56–64).

Agonists of ACE2 or inhibitors of ACE/Ang2, are being explored as treatments for COVID-19 patients (10), as they would function to maintain the balance of the RAS-KKS system and reduce further inflammation (70). Of note, Khan et al. conducted a phase II trial in 10 critical care patients with ARDS utilizing a recombinant ACE2 agonist to reduce lung injury. Despite the expected effect in the RAS system, decreasing the angiotensin II level, the study was stopped early because oxygenation and lung compliance did not differ between groups (73). Additionally, there was an unexpected increase in surfactant protein D (SP-D) serum levels, which when present in the blood marks increased capillary leakage (73). Collectively, these observations suggest that ACE2 agonist or ACE inhibitors should be pursued with caution.

### Systemic Inflammatory Response Syndrome

As described above, the loss of epithelial protection from the glycocalyx and the increase in permeability factors (e.g., VEGF) in the lung endothelial typical of SARS, might lead to the release of the virus and other PAMPS from the lungs to the blood stream (26). Consistently, there are reports of SARS-CoV-2 mRNA present in stool, blood and urine in severe COVID-19 patients (52). In contrast, a study in nine SARS-CoV-2 infected patients from the onset of infection until after the resolution of flu-like symptoms, showed no viral RNA was found in the blood or urine (74).

The systemic release of TNF-α, IL-1β, and IL-6 can impact many organ systems, consistent with the multi-organ effect observed in COVID-19 (18, 75). As mentioned above, alveolar macrophages and airway epithelial cells are expected to be the major source of those cytokines during COVID-19. Those cytokines can hyperactivate monocytes/macrophages and T lymphocytes and result in CSS and MAS/sHLH (20). More details of the CSS consequences can be found in a perspective article by Moore and June (18). In one study reporting on 54 COVID-19 patients, 28 of those patients had severe respiratory failure and all of them displayed either MAS or low HLA-DR, with lymphocytopenia (17). Interestingly, up to 50% of patients with systemic juvenile idiopathic arthritis and MAS/HLH present nervous system involvement, ranging from mild confusion to coma (76). Moreover, evidence of neuropathology has been reported during COVID-19, including 26 out of 40 patients noted to have confusion according to the Confusion Assessment Method for the ICU (77). Given the apparent contribution of MAS to progressing severity of COVID-19, treatment with bisphosphonates provide a potential added benefit, although we could not find studies addressing the potential effect of bisphosphonates on MAS.

Bone marrow stimulation by acute lung injury derived pro-inflammatory cytokines leads to the release of immature granulocytes in the blood circulation (45). As adhesins are upregulated in the lung endothelium, leukocyte infiltrate will be present. Of note, leukocytes and immature leukocytes have been reported in other lung injury scenarios to sequester in the lung microvasculature where they contribute to tissue pathology and inflammation (26). Sequestration of blood leukocytes may result in low white blood cell count, which has been reported in severe COVID-19 patients (49, 55, 78). CD8 T cells are classical antiviral responders, thus treatments targeting activation of lymphocytes are likely to be protective early in the infection. An updated meta-analysis by Soraya and Ulhaq define crucial laboratory parameters that can be used in COVID-19 diagnosis and prognosis, indicating that higher leukocyte counts, neutrophils, D-dimer, and CRP positively correlates with severe COVID-19 when compared to non-severe cases (79). As CD8 and CD4 T cells from COVID-19 patients do not express high levels of exhaustion markers when in circulation, the investigation of adequate windows of treatments targeting those cells is essential (80).

Another important aspect of the bone marrow stimulation is the increase in blood coagulability, which is a major problem for patients with comorbidities. Lung derived IL-6 stimulates hepatocytes to produce acute phase proteins, such as CRP, fibrinogen and antiproteases, stimulating hematopoiesis, specifically the production of platelets (26). Platelets, leukocytes and erythrocytes can shed microparticles (MP) associated with lung injury, and have been reported in collagen vascular disorders. MP are known to be involved in inflammation, coagulation, and are increased in atherothrombotic cardiovascular diseases (81). Reports of periphery and organ clotting are increasing in COVID-19 clinical description. It is noteworthy that systemic increased endothelial adherence and permeability, reduced NO, higher number of platelets and monocytes, higher production of ROS and lipid content, higher proteinase activity, and lower fibrosis cap of plaques might result in increased heart attacks and strokes in those patients (25). Recently, there was a report of large-vessel stroke in five patients younger than 50 years of age in NY (82). Consequently, anticoagulants and regulators of RAS-KKS might work at preventing clots and further vascular problems. Of note, they have been used in coagulopathy with some degree of success for COVID-19 (48). Many reports of coagulation complications due to COVID-19 are happening after discharge of patients. It seems that cytokine storm presentation is not needed for the release of pro-thrombotic factors (repair attempt) after CALI stimulation of the hepatocytes and bone marrow, and the opposite is also true. As D-dimers are a subproduct of inflammation triggered during sustained acute phase response, it is of essence to target upstream cascades of inflammation using antivirals, bisphosphonates, and anti-inflammatory treatments in a timely manner.

### Systemic Failure

When vascular permeability and systemic inflammation are too high, vascular dysfunction, and inflammation might lead to multiple organ failure (MOF), including respiratory failure (25). Compassionate treatments are trying to address many inflammatory aspects of the disease presentation (18). For example, the anti-IL-6 antibody, Tocilizumab (TCZ) has been used to dampen the inflammatory response. Reduction of IL-6 might impact thrombus resolution, as reported in mice (83). Differently, TCZ has shown some success at reducing Cytokine Release Syndrome (CRS) during CD19 CART-cell therapy (84). Accordingly, different studies using TCZ presented increased likelihood of survival in patients with severe COVID-19 (85–88). Another group reported that the earlier the treatment of COVID-19 with TCZ, the better the likelihood of survival (89). Anakinra, a recombinant IL-1 receptor antagonist, reduced both need for MV in the ICU and mortality among patients with severe COVID-19, in different cohort studies (90–94). Controlled trials are still required.

As suggested by our proposed model, prevention of the second-hit, or preventing the amplification of lung-systemic inflammation loop, seems to be the best window for intervention specially in patients with comorbidities. Although immunomodulatory treatments will dampen aspects of the pro-inflammatory response, the epithelial antiviral type I IFN response will still be in effect, providing the immune system with a mechanism to clear the SARS-CoV-2 infection. Importantly, immunomodulatory drugs should be helped by the use of antivirals whenever viral particles are still detected.

Coagulopathy appears to be critical in the context of COVID-19. One study reported procoagulant presences even in early stages of COVID-19 (47), consistent with RAS-KKS unbalance influencing the production of pro-thrombotic factors (11). The collective coagulation problems accumulated in the other phases of COVID-19 likely result in the observed high levels of fibrin degradation product, D-dimer, longer prothrombin time, and activated partial thromboplastin time in critically ill patients compared to survivors (95). As noted above, systemic acute phase response by the liver and bone marrow produce the bulk of pro-thrombotic factors. It is noteworthy that microthrombosis are compatible with increased severity of hypoxemia maintaining lung compliance. Finally, other exacerbated immune response factors are likely contributing to the coagulopathy, such as inflammasome activation, NETs, endothelial cell infections and pyroptosis.

We advocate an individualized approach to mechanical ventilation (MV) parameters based on patient's lung mechanics as a support therapy. The current mortality rate of patients under MV is alarmingly high (32, 96). Many patients in need of MV arrive at the ICU with systemic hyperinflammation, likely resulting in quicker deterioration. Moreover, inadequate ventilation settings might exacerbate lung injury and further contribute to lung-systemic inflammation. Thus, individualized care and drug therapies to prevent this feed-forward loop between lung-systemic inflammation leading to MOF and coagulopathy are urgently needed.

## Summary of What Might Be Happening After CALI in Severe COVID-19

**Figure 2:**
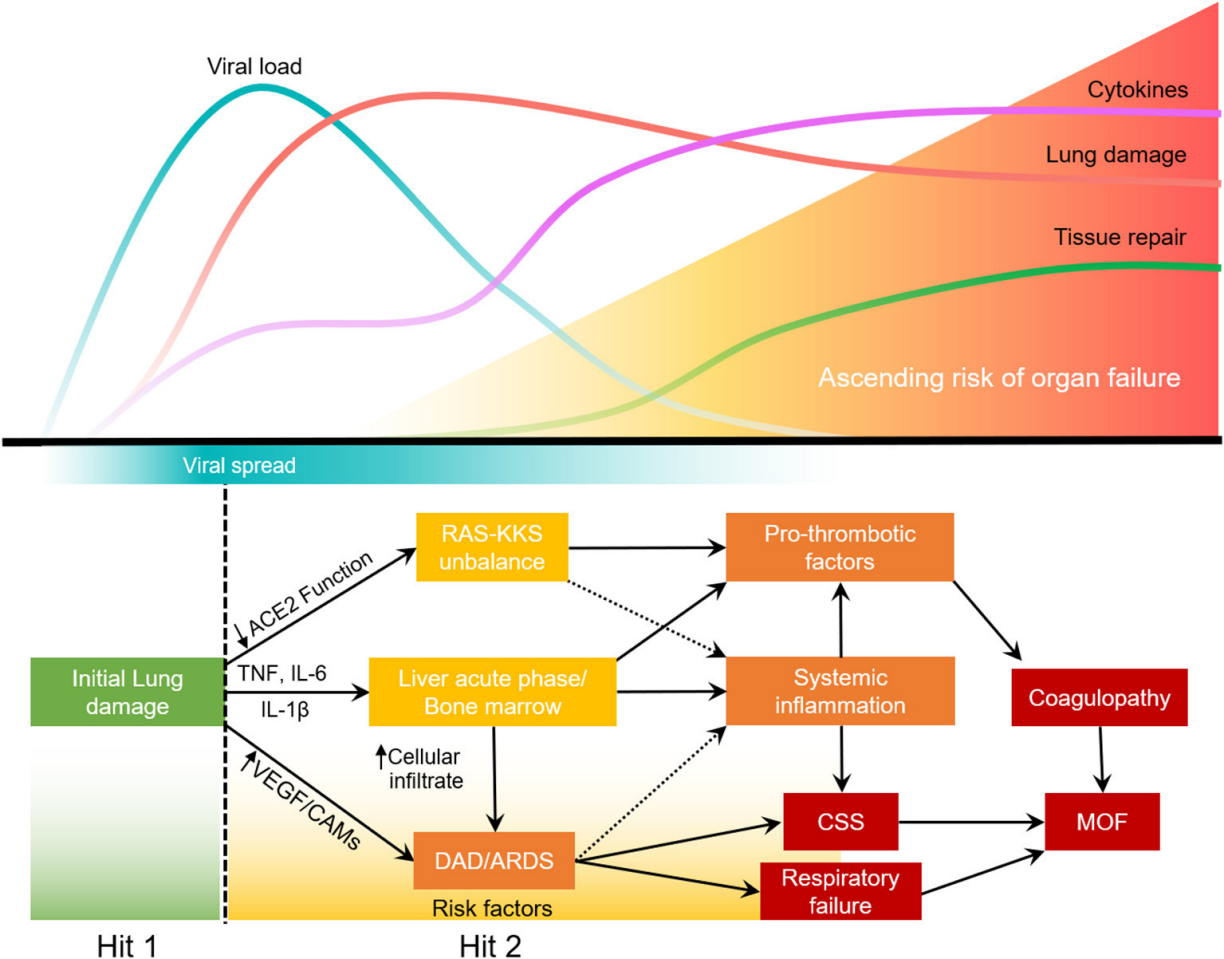
Two-hit model of systemic inflammation derived from COVID-19 associated lung injury.

Most SARS-CoV2 infections will not develop into severe COVID-19. Upon lung injury (first hit), the host response will determine the severity of the disease (35–37). In some patients, lung infection leads to the activation of alveolar macrophages and lung epithelial cells that will release proinflammatory cytokines. IL-6, TNF, and IL-1β increase expression of cell adhesion molecules (CAMs) and VEGF in the lung, thereby increasing permeability of the lung endothelium and reducing barrier protection, allowing viral dissemination and infiltration of neutrophils and inflammatory monocytes. In the blood, these cytokines will stimulate the bone marrow to produce and release immature granulocytes, that return to the more adherent lung endothelium and further increase lung inflammation, leading to ARDS. This second hit in the lung leads to a loop of systemic inflammation resulting in cytokine storm syndrome. Concurrently, the acute phase response caused by the stimulation of hepatocytes and bone marrow increases the production of platelets, fibrinogen and other pro-thrombotic factors. The systemic decrease in ACE2 function due to virus infection, impacts RAS-KKS that culminate in increased clotting and inflammation. The disbalance of the RAS-KKS is common in patients with comorbidities, and affects the lung, kidneys, liver, brain and the gastrointestinal system (97). All of which culminates in respiratory failure, coagulopathy and potential multiple organ failure (MOF) (Figure 2).

## Further Considerations between COVID-19 and Systemic Inflammatory Response Induced by Lung Inflammation

The exacerbated inflammatory response to acute lung injury is more frequent in males and increases with age, being very low in young individuals (15–19 years of age), whereas mortality can reach up to 60% in individuals above 85 years of age (98). Consequently, we suspect age-dependent responsiveness to lung injury may contribute to the age-disparity of severe COVID-19, with children under the age of 20 rarely developing severe disease (25, 49). Other possibilities that may contribute to protection of children from COVID-19 are frequent infection with mild viruses and bacteria, vaccinations in their first decade of life, and the lack of chronic underlying conditions observed in the COVID-19 high risk groups. All of these likely contribute to high levels of IgA in the upper respiratory tract, which may confer cross-protection against SARS-CoV-2, coupled with high local (or systemic) IFN-γ which could potentially reduce the COVID-19 entry and multiplication success in younger individuals. This protection may also be associated with “trained immunity” (99), which is consistent with the prospect of using BCG vaccine as a preventative treatment for SARS-CoV-2.

The regulation of the RAS system for COVID-19 has been discussed from the cardiovascular viewpoint by Guo et al. (100). A positive correlation between Angiotensin II and lung SARS-CoV-2 viral load was described by Liu et al. (101). There have been many attempts to neutralize the lung inflammatory components using “imunoresolvents” for SIRS-ALI with promising preclinical testing, such as for COVID-19. Unfortunately, in an analysis between 22 preclinical and clinical sepsis trials, these drugs failed to show benefit (102). It is noteworthy that other randomized double blinded studies using infliximab (anti-TNF-α), had no therapeutic benefit reducing acute exacerbation of chronic obstructive pulmonary disease (103). Statins represent another class of drugs with potential to reduce inflammation and increase ACE2 expression (104). Pro-resolving lipid mediators including resolvins, protectins, and maresins have anti-inflammatory activity in lung inflammation and might help in a treatment cocktail for COVID-19 (26). The reasons why we think that a cocktail of treatments targeting multiple aspects of this systemic presentation due to lung injury is represented by the work of Cao et al. showing that the antivirals Lopinavir-Ritonavir failed to reduce COVID-19 lethality, although it reduced the ICU time of recovered patients by 5 days (105).

Antivirals coupled with bisphosphonates might be able to impede progression of COVID-19 to severe and critical. Antivirals combined with regulation of the causes for coagulation and vascular dysfunction, thereby preventing the lung from amplifying systemic inflammation due to mechanical ventilation, comorbidity, or secondary infection might also be of essence to reduce lethality and increase recovery rate in COVID-19. Finally, as proposed by several groups, antivirals coupled with drugs addressing CSS can be also beneficial to prevent COVID-19 progression (20, 106).

## Author Contributions

RP: conceptualization and writing—original draft. RH, RS, and NS: writing—review and editing. NS: supervision. All authors contributed to the article and approved the submitted version.

## Conflict of Interest

The authors declare that the research was conducted in the absence of any commercial or financial relationships that could be construed as a potential conflict of interest.
